# Socio-cultural, environmental and behavioural determinants of obesity in black South African women

**DOI:** 10.5830/CVJA-2013-069

**Published:** 2013-11

**Authors:** Lisa K Micklesfield, Estelle V Lambert, David John Hume, Sarah Chantler, Paula R Pienaar, Kasha Dickie, Julia H Goedecke, Thandi Puoane

**Affiliations:** MRC/Wits Developmental Pathways for Health Research Unit, Department of Paediatrics, Faculty of Health Sciences, University of the Witwatersrand, Johannesburg, South Africa; UCT/MRC Research Unit for Exercise Science and Sports Medicine, Department of Human Biology, UCT School of Health Sciences, University of Cape Town, South Africa; UCT/MRC Research Unit for Exercise Science and Sports Medicine, Department of Human Biology, UCT School of Health Sciences, University of Cape Town, South Africa; UCT/MRC Research Unit for Exercise Science and Sports Medicine, Department of Human Biology, UCT School of Health Sciences, University of Cape Town, South Africa; UCT/MRC Research Unit for Exercise Science and Sports Medicine, Department of Human Biology, UCT School of Health Sciences, University of Cape Town, South Africa; UCT/MRC Research Unit for Exercise Science and Sports Medicine, Department of Human Biology, UCT School of Health Sciences, University of Cape Town, South Africa; UCT/MRC Research Unit for Exercise Science and Sports Medicine, Department of Human Biology, UCT School of Health Sciences, University of Cape Town, South Africa; UCT/MRC Research Unit for Exercise Science and Sports Medicine, Department of Human Biology, UCT School of Health Sciences, University of Cape Town, South Africa; South African Medical Research Council, Parow, South Africa; School of Public Health, University of the Western Cape, South Africa

**Keywords:** South Africa, obesity, food security, diet, physical activity, body image, socio-economic status

## Abstract

**Abstract:**

South Africa (SA) is undergoing a rapid epidemiological transition and has the highest prevalence of obesity in sub-Saharan Africa (SSA), with black women being the most affected (obesity prevalence 31.8%). Although genetic factors are important, socio-cultural, environmental and behavioural factors, as well as the influence of socio-economic status, more likely explain the high prevalence of obesity in black SA women. This review examines these determinants in black SA women, and compares them to their white counterparts, black SA men, and where appropriate, to women from SSA. Specifically this review focuses on environmental factors influencing obesity, the influence of urbanisation, as well as the interaction with socio-cultural and socio-economic factors. In addition, the role of maternal and early life factors and cultural aspects relating to body image are discussed. This information can be used to guide public health interventions aimed at reducing obesity in black SA women.

## Prevalence of obesity

According to the World Health Organisation (WHO), obesity is a global epidemic that affects 500 million people worldwide, and is predicted to increase to one billion people by 2030.^1^ The rising prevalence of obesity is associated with an increased risk of non-communicable diseases (NCDs), such as cardiovascular disease, type 2 diabetes and several types of cancer.^2^

Until recently, Africa has been spared from this epidemic as it grappled with under-nutrition, as well as infectious diseases such as HIV and tuberculosis. However, over the last century the continent has seen a rapid rise in the prevalence of overweight and obesity, and their associated co-morbidities.^3^-^5^ This dual burden of disease in Africa is particularly devastating as it is compounded by the metabolic consequences of the roll out of anti-retroviral medications in certain countries.^6^,^7^

Within sub-Saharan Africa (SSA), the prevalence of obesity differs widely from as low as 1% in Ethiopia^8^ to as high as 27% in South Africa (SA).^9^ Only three other countries in SSA report a national obesity prevalence of over 20%, including Mauritania (23.3%),^10^ Swaziland (23.1%)^11^ and Gabon (21.5%).^10^

In SA, statistics from the 1998 National Demographic and Health survey (SADHS) reported an obesity prevalence of 30% in all women over the age of 15 years, which is more than three times higher than the prevalence in men (7.5%).^12^ Those most affected were black women, with a prevalence of 31.8%, compared to 6% in black men, 22.7% in white women, 21.1% in Indian women and 26.3% in women of mixed ancestry.

The most recent SADHS undertaken in 2003^9^ reported that the prevalence of obesity remains high in black women (28.5%). Of concern is the large increase in the prevalence of obesity among black SA adolescent girls,^13^ who will soon be entering adulthood and will therefore be at increased risk for future NCDs.

**Fig. 1.**
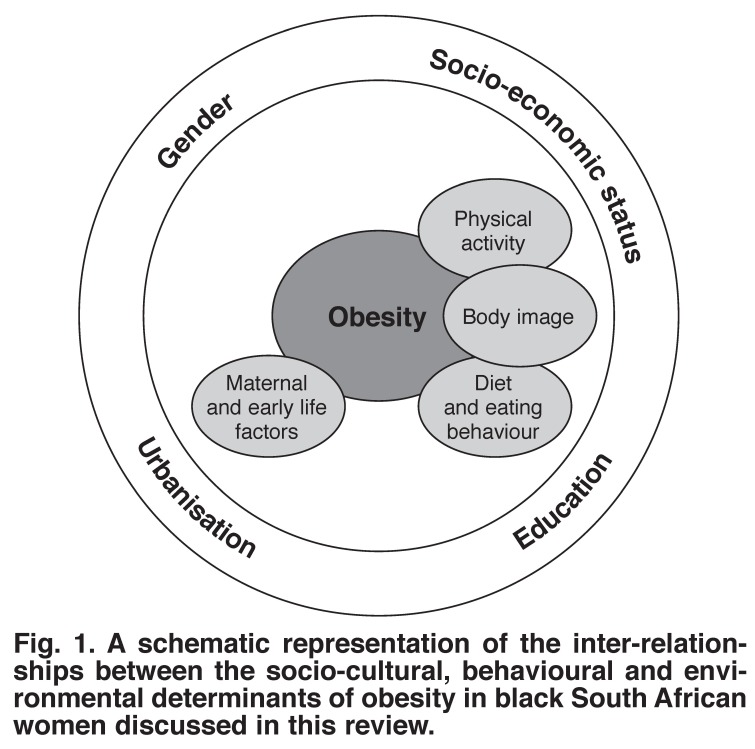
A schematic representation of the inter-relationships between the socio-cultural, behavioural and environmental determinants of obesity in black South African women discussed in this review.

For the purposes of this review, we attempted to outline the socio-cultural, environmental and behavioural determinants of obesity in black SA women, and compare them to their white counterparts, black SA men, and where appropriate, to women from SSA. The literature included in this review was selected from the available literature to highlight the magnitude and complexity of the determinants of obesity in this population (Fig. 1).

## Definitions of race, ethnicity and culture

To contextualise this review, it is necessary to consider our definitions of race, ethnicity and culture, and the potential interactions between these constructs, particularly within the SA context where there are the potentially confounding effects of socio-economic status. Race has been defined by Williams *et al.*^14^ as ‘a complex multidimensional construct reflecting the confluence of biological factors and geographical origins, culture, economic, political, and legal factors, as well as racism’.

In a recent commentary,^15^ it was suggested that race and ethnicity share a similar definition, however, the difference between the two constructs lies in the fact that ethnicity is usually defined by the group itself, whereas race is typically defined by others outside ‘the group’. Culture, on the other hand, has been defined as ‘the learned and shared beliefs, values, and life ways of a designated or particular group which are generally transmitted inter-generationally and influences one’s thinking and action modes’.^15^ For demographic and restitution purposes, the Government currently classifies race into black (ethnic Africans), white (Europeans, Jews and Middle Easterners), coloured or mixed ancestry (mixed race) and Indian (South Asian).

## Socio-economic status and education

Historically, black South Africans have been compromised in terms of education, access to healthcare and earning capacity under apartheid laws. This is still currently reflected in the 2008/2009 South African Living Conditions of Household Survey (LCS),^16^ in which it was demonstrated that 25% of black households fell within the lowest quintile of annual household consumption expenditure compared to 0.7% of white households, whereas 81% of white households fell within the highest quintile compared to 8.2% of black households. Differences in obesity and disease prevalence between these ethnic groups may be partly attributed to or mediated by these social inequalities.^17^

Studies in developed countries have shown an inverse relationship between socio-economic status and obesity,^18^,^19^ however studies in SA,^12^,^20^-^22^ as well as other SSA countries^23^-^26^ show a consistent positive association between obesity and socio-economic status. In these studies, obesity was positively associated with access to clean water and electricity,^21^,^25^ reduced housing density,^22^,^25^ as well as more money spent on food,^27^ higher energy intake,^25^ commuting by taxi/vehicle^28^ and reduced physical activity or increased sedentary behaviour,^22^,^28^-^31^ factors representing a transition towards a more Western lifestyle. In addition, in many black African communities, obesity or overweight may still be considered a sign of good health and beauty, as well as affluence,^32^,^33^ further impacting on the relationship between socio-economic status and obesity.

On the other hand, level of education, although highly related to socio-economic status has been shown to be independently associated with obesity in SA and other SSA countries. Studies in many SSA countries,^23^,^24^,^27^ as well as regions in SA with lower socio-economic status,^21^ have shown a positive association between level of education and obesity.

By contrast, results from the SADHS suggest that the relationship between education and obesity is not linear, as women with no education and women with a tertiary education had a lower body mass index (BMI) than those with some schooling.^12^ This may reflect the wider distribution of both education and socio-economic status in SA, which has recently been re-classified as a middle-income country,^1^ and which has one of the highest GINI coefficients in the world, suggesting extreme inequality with regard to poverty and wealth.^34^

## Gender

Studies in SA^12^,^20^,^21^,^35^ and other SSA countries^23^,^25^,^26^,^28^,^29^ have consistently reported that the prevalence of obesity is greater in women than men. Case and Menendez,^20^ using data collected from an informal urban settlement in SA, identified two factors to explain the gender difference in obesity rates in their study: (1) being nutritionally deprived as children; and (2) having a higher socio-economic status. These factors were associated with obesity in women, but not in men.

Traditionally, black SA households are strongly patriarchal, with men holding a dominant position. For this reason, boys have been better cared for and nourished as babies and infants, so they do not necessarily experience the same level of nutritional deprivation at a young age as girls.^36^ However, due to migrant labour and high death rates related to HIV/AIDS among young adults, nearly half of all households in SA are headed by women.^37^ These households are among the poorest and most marginalised.^16^ In 2009, more than 20% of female-headed households reported experiencing hunger (skipping meals or running out of money) compared to only 15% of male-headed households.^38^

## Urbanisation

Rural and urban black SA communities have historically faced very different public health challenges, with infectious diseases associated with under-nutrition prevalent in rural communities, and a rising prevalence of NCDs associated with over-nutrition in urban-dwelling communities.^30^ This rural–urban gradient is still present in most SSA countries,^30^ but in SA, the disparities between rural and urban settings are attenuated. The 2003 SADHS reported a 21% prevalence of obesity in rural black SA women compared to 31% in urban black SA women.

Urbanisation is accompanied by the adoption of a Westernised lifestyle, however in SA many cultural beliefs around lifestyle behaviours and body image are retained.^36^ Differences in diet have been identified as one of the possible causes of urban–rural differences in obesity prevalence,^39^ and the term ‘nutrition transition’ is now commonly used to refer to changes in the diet that occur with urbanisation.

Recent data also suggest however that the nutrition transition is occurring within rural areas, possibly explaining the increasing prevalence of obesity in less developed settings.^40^-^42^ Another major contributing factor to the high prevalence of obesity in urban versus rural communities in SA and SSA is the increase in physical inactivity and the adoption of a more sedentary lifestyle with urbanisation.^30^,^31^,^43^

## Maternal and early life factors

Nutrient deprivation and the timing thereof during the intrauterine period leads to foetal programming, resulting in genetic and epigenetic adaptations.^44^,^45^ These biological adaptations predispose an individual to obesity when exposed later in life to an environment abundant with energy-dense and/or high-fat foods, as is currently experienced in middle-income countries such as SA.^46^

The prevalence of low birth weight (< 2.5 kg), very often the consequence of nutrient deprivation in utero, is 15% in SA, which is marginally higher than the overall prevalence of 13% in SSA.^47^ The COHORTS initiative, a study of birth cohorts in five low- or middle-income countries including SA, has shown that size at birth is linked to major features of the metabolic syndrome in adulthood, including obesity.^48^,^49^ However the relationship between pre-natal exposure and obesity in later life has been shown to fit a U-shaped curve. More specifically, low birth weight has been associated with increased levels of adult abdominal adiposity, while high birth weight was associated with overall adult adiposity.^50^,^51^

High birth weight has been shown to be a result of excessive maternal body weight or excessive weight gain during pregnancy.^52^,^53^ This is of concern in SA, given the high prevalence of obesity in SA adolescents and adult women. It is compounded further by healthcare inequalities, associated perceptions of the healthcare system, and the periodic lack of adequate resources that have led to late or poor attendance rates at antenatal clinics.^54^,^55^

Under-nutrition during the first six months of life increases the risk of stunting. Global statistics indicate that in SSA, the prevalence of stunting under the age of five years is 39%, with stunting rates ranging from 27% in Ghana to 55% in Niger, and SA reporting a stunting prevalence of 24%.^47^ In transitional societies of SSA, stunting and adolescent obesity may co-exist in the same geographic population.^56^

A cross-sectional growth survey conducted in rural SA children and adolescents aged one to 20 years showed that an estimated one in five children aged one to four years was stunted. Concurrently, the prevalence of combined overweight/obesity was 20–25% among girls in late adolescence.^56^ Steyn *et al.* showed that stunting in children under the age of nine years resulted in a 1.8-fold increased risk of obesity.^57^ Moreover, other evidence suggests that individuals who were stunted as children were more likely to be overweight as adults.^58^ Furthermore, excessive weight gain during childhood was associated with adult body composition.^59^

## Physical activity

Physical activity may be defined as any bodily movement produced by skeletal muscle that requires energy expenditure.^60^ Prior to the early 2000s, the evidence base for physical activity and health in SSA was limited, fragmented and localised, with few nationally representative samples. Self-report physical activity questionnaires were not standardised, often not validated in the populations in which they were being applied, and the focus was primarily on energy balance and seasonal agriculture-related physical activity and under-nutrition.

Recent WHO Stepwise surveillance initiatives, using a common instrument called the Global Physical Activity Questionnaire (GPAQ), have yielded a growing body of evidence on the global trends in physical activity and inactivity.^61^ The physical activity recommendations for health in adults are defined as engaging in at least 150 minutes of moderate-intensity activity per week, or 75 minutes of vigorous-intensity activity per week, or an equivalent combination of moderate- and vigorous-intensity activity.^62^ Physical inactivity has been defined as ‘doing no or very little at work, at home, for transport or during discretionary time’.^63^

In the African region, estimates of the prevalence of inactivity are widely varying, ranging from as low as 3.8 and 1.5% in women and men in the Comoros, to 15 and 9% in Ghanaian women and men, and 48 and 45% in SA women and men, respectively.^61^,^63^,^64^ The highest reported prevalences of inactivity in this region are similar in magnitude to those seen in North America, and higher than those reported in South America, Western Pacific or Asia.^10^

It appears that the inactivity gradient and obesity seem to be related to development within the region and within the country. An ecological evaluation of inactivity in women and men in 13 SSA countries demonstrated a significant correlation between gross national income (per capita) and prevalence of selfreported inactivity.^65^

Sobngwi *et al.*^66^ studied over 1 600 Cameroonian adults living in either rural or urban settings and found that lowered the odds for overweight and obesity in a dose-dependent manner, and that the odds for overweight and obesity, as well as impaired glucose tolerance, were significantly increased with increased lifetime exposure to an urban environment (percentage of life in a city). Conversely, in SA, results from the THUSA study showed that among a group of black adult women, physical inactivity was a stronger correlate of obesity than socio-economic status and dietary factors.^31^ As physical activity has been identified as playing a key role in influencing health outcomes, even in communities undergoing epidemiological transition, trends in physical activity behaviour have implications for public health and the emerging burden of NCDs in the region.

Armstrong and Bull^67^ highlighted that in developing countries, ‘occupational-, domestic- and transport-related activities may contribute more to overall energy expenditure than leisure-time or recreational activity’, and therefore a multi-domain approach to the measurement of physical activity is essential. A recent study including data from 22 African countries showed a higher proportion of adult men compared to women (84 vs 76%) meeting the global physical activity recommendations.^64^ Although levels of physical activity varied greatly across these countries and population sub-groups, the study found that leisure time activity (5%) was consistently low, irrespective of gender, whereas work activity (moderate and vigorous combined) contributed the most (49%) to total physical activity time, followed by transport-related activity (46%).^64^

In SA, 25–37% of adults are sufficiently active,^63^ and data from the 2003 SADHS has shown that half the population are insufficiently active.^9^ Moreover, the SA survey shows a rural-to-urban gradient, with reduced physical activity levels with increasing urbanisation. Moreover, increasing education is associated with reduced occupational physical activity and increased leisure activity. These findings are corroborated by objective measurement in smaller regional studies in SSA, which demonstrate similar physical activity trends, with adult men being more physically active compared to adult women in both urban and rural settings, and education level affecting the domain of activity.^68^-^70^

Traditional methods for collecting physical activity by selfreport may over- or under-estimate actual levels.^71^ Moreover, ‘light activity’ is overlooked entirely. This is despite the fact that urban-dwelling persons in low- or middle-income countries such as SA are likely to spend a relatively large portion of their day in at least light activity, as opposed to being entirely sedentary (Kroff, pers commun, 2012). Importantly, work by Cook *et al.*^43^ has demonstrated that even light activity (accumulated steps per day) is associated with a reduced risk for obesity in a dosedependent manner. Adjusting for age, motor vehicle access, education, tobacco use and co-morbidities, and BMI was 1.4 kg/m^2^ lower per 5 000 steps/day, and compared to being sedentary, the risk of obesity (BMI ≥ 30 kg/m^2^) was 52% lower for 10 000 steps/day.

In countries such as SA, factors such as culture, socio-economic status and the built environment may act as barriers to physical activity. For example, in a convenience sample of largely urban-dwelling South Africans, self-reported leisure-time moderate to vigorous physical activity was significantly higher in those persons living in neighbourhoods in which crime was not perceived to be a problem. These results are supported by recent work from Nigeria where they showed that perceived safety, aesthetics and cleanliness were inversely associated with obesity and positively associated with physical activity.^72^

However, in SA communities, walking for transport has still been shown to be higher in persons from communities in which there are no pavements (Lambert, Tshabangu and Naidoo, pers commun, 2012), suggesting that many behaviours are outside of an individuals own volition. Cultural barriers to physical activity in black SA women include the acceptability of wearing tight-fitting clothing when participating in sport, as well as the perception that participating in leisure-time physical activity takes time away from household chores.^73^

## Diet and eating behaviour

Dietary intake and quality have been shown to be associated with the prevalence and risk of obesity.^74^ Obesogenic dietary behaviours include a high-energy intake, high dietary fat and sugar intake, low-fibre fruit and vegetable intake, or a combination of the above. Several of these dietary habits and behaviours are associated with the adoption of a more Western lifestyle and represent the nutrition transition in developing countries.

When compared to other SSA countries, SA is considered to be further along the nutrition transition, characterised by higher intakes of dietary energy (600 kCal above the mean for 39 other SSA countries) and fat intake (24.5% vs sample mean of 18.9%), as well as higher levels of obesity than other countries.^46^ In a study of Kenyan and SA women, Steyn *et al.*^75^ showed that the rural environment differed between countries, with more than 60% of rural Kenyan women having access to land, which was associated with a higher nutrient mean adequacy ratio, dietary diversity score and food variety score than rural SA women. This finding highlights the difference in the effect of the rural–urban environment of different populations along the nutrition transition.

In SA, data from the 2003 SADHS showed an increase in dietary quality with urbanisation, as characterised by an improvement in micronutrient intake (micronutrient score based on tertiles of the RDA).^9^ In contrast, Oldewage-Theron *et al.*^76^ reported that nutrient quality was poor in peri-urban black SA women, with low food variety and diversity scores attributed to low household food security and availability. Consistent within all of these SA studies, including the THUSA study,^39^ urbanisation was associated with an increase in dietary fat intake, which corresponded to the increased prevalence of obesity in urban compared to rural women.

Most black South Africans who urbanise do so into informal settlements that may not be situated close to any of the large food chains that offer a greater variety and quality of food. The most convenient place to purchase food is from informal vendors who sell inexpensive and less varied foods of poor quality. Indeed, data from a study in SA children showed that the lack of grocery-style shops and many accessible tuck shops and street vendors is shaping new buying habits of children that include a higher intake of less nutritious foods.^77^

For example, a study of adolescents in the same cohort reported the frequent purchase of the ‘quarter’, a combination of white bread, polony, fried chips and cheese, as a meal.^78^ The ‘quarter’ is of good economical value based on the cost/kCal, but is low in fibre and micronutrient quality. Temple *et al.* have shown that a healthy diet is more expensive than a less healthy diet, and therefore is not affordable for the majority of South Africans.^79^

Socio-economic status is another important factor that influences dietary quality and food choices. Increased wealth and disposable income contribute to food choices and are associated with the aspiration to consume more meat products, bigger portion sizes, and a more frequent intake of fast foods.^73^ Conversely, low household food security is associated with poor dietary quality, characterised by low food variety and diversity scores.^80^

Household food security may be described as a continuum, from food secure, food insufficiency (some concern regarding having enough funds to buy food for the month, without changing diet), low food security (typically reducing the quality of the diet), to food insecure (where there is a reduced food intake and skipping meals).^81^ Notably, mothers who are food insecure are more likely to be overweight or obese than men and women without children, and food-insecure fathers.

Martin and Lippert^81^ showed that this is not as a result of biological changes that occur with pregnancy, but rather may be the adoption of strategies, albeit unhealthy, to protect their children when faced with food insecurity. Furthermore, single mothers appear to be at greater risk for food insecurity and obesity, compared to women with partners. However, once households are truly food insecure, women are more likely to be underweight.

In low-income countries in SSA, children of overweight mothers are often underweight,^82^ which differs from the situation in SA in which children of overweight mothers are more likely to be overweight.^83^ The notion that food insecurity is implicated in adult obesity is paradoxical, but may be explained by the consumption of energy-dense foods of low nutritional value.

## Body image

Cross-sectional studies have revealed that, unlike the vast majority of women who favour the lean, Westernised archetype, there is a preference for a larger body size among black SA women.^32^ This ideal stems from a cluster of culture-bound beliefs, which promote lifestyle behaviours commonly associated with obesity.

International research has consistently shown that, after controlling for age, education, socio-economic status and body weight, men, irrespective of ethnicity, and black women display the lowest degrees of body size dissatisfaction compared to other ethnic groups.^84^-^87^ Furthermore, results from the SADHS confirm that black women were more likely to under-estimate their body size compared to women of other ethnic groups.^12^ In addition to black SA families showing a greater tolerance for increased body size,^33^ strong mother–daughter resemblances have been identified for numerous body image constructs, including body size ideals and perceptions of body size dissatisfaction.^88^

Socialisation moulds the body image of these women throughout all life stages, and may explain why this ideal is so well maintained from early childhood into adulthood. For instance, young girls are encouraged to be plump, with weight gain prior to marriage indicative of fertility and the ability to bear children.^89^ In addition, while men are socialised to do hard labour, girls are expected to perform light labour, which may provide limited motivation for a leaner body since activities of this nature do not necessitate high levels of physical aptitude.^90^

Similarly, low physical activity is due to the belief that physical activity is associated with weight loss, as well as sub-optimal environmental conditions such as a high crime rate and overcrowding.^12^,^32^ Notably, similar attitudes toward weight control have been found among black women in rural areas, where it was shown that most overweight and obese women did not desire weight loss.^91^

Ethnic body size preferences have been shown to govern how individuals respond to insults such as disease and sexual abuse. For example, a widely held belief among black SA women is that large people are happy and healthy, whereas those who are slender are perceived to experience personal problems and that they may have diseases such as HIV/AIDS.^92^

Furthermore, Goedecke *et al.*^93^ demonstrated that ethnicity altered the relationship between childhood sexual abuse and obesity. In this small study, white women who were sexually abused as children were more likely to be obese as adults. As obesity is viewed as less attractive, this has been suggested to protect against future sexual advances/abuses. By contrast, black women who were sexually abused as children were more likely to be lean, which was suggested as a means of protecting themselves from further abuse. Furthermore, other studies have reported that large women are respected, dignified and cannot be pushed around.^32^,^94^

The influence of family and community also alters body size and satisfaction. For example, once a woman marries, she is encouraged to gain weight as this signifies her husband’s ability to support her financially.^32^ In addition, the mother of the household is expected to be an authoritative figure capable of commanding respect from her children.^36^ This, combined with the expectation by black SA communities that people in positions of power should be big, promotes the adoption of higher degrees of tolerance for an increased body size. Puoane *et al.*^32^ conducted a study on SA community health workers, who are respected and important members of the community and who play an important role in assisting with the communication between the community and the formal health sector, and found that 95% were overweight or obese.

Given that media influences extend further into disadvantaged areas as the economy improves, black SA women are increasingly exposed to conflicting body size ideals. Future studies should therefore monitor the effect of such influences on body size preferences.

## Conclusion

There is compelling evidence that the prevalence of obesity is increasing in SSA, and that this increase is linked to urbanisation, economic development and concomitant lifestyle risk factors, such as physical inactivity and poor dietary practices. In addition, there are a number of paradoxes that have emerged, including the positive association between food insecurity and obesity, the non-linear association between education and obesity, as well as the distinct differences between patterns and determinants of obesity in men and women in the region.

Although this was not a systematic review, which may be considered a limitation, this review highlights the complexity of various socio-cultural, environmental and behavioural factors associated with obesity in black SA women. Public health interventions targeted at individual behavioural risk factors, although important, may have limited success in reducing obesity if other contributing factors such as culture, environment and socio-economic status are not considered.

## Key messages

• The prevalence of obesity is increasing in SSA, and is linked to urbanisation, economic development, and concomitant lifestyle risk factors, such as physical inactivity and poor dietary practices.

• Socio-cultural, environmental and behavioural factors, as well as the influence of socio-economic status, contribute significantly to the high prevalence of obesity in black SA women.

• Barriers to physical activity in black SA women include culture, socio-economic status and the built environment.

• Food insecurity and dietary quality contribute to the prevalence of obesity in SA.
